# An exploration of adherence to the World Health Organization’s Prosthetic Standards in Namibia

**DOI:** 10.4102/ajod.v14i0.1614

**Published:** 2025-06-25

**Authors:** Surona J. Visagie, Christopher M. Likando

**Affiliations:** 1 Division of Disability and Rehabilitation Studies, Faculty of Medicine and Health Sciences, Stellenbosch University, Cape Town, South Africa

**Keywords:** Namibia, personnel, products, prosthetics, provision, policy, World Health Organization standards

## Abstract

**Background:**

The World Health Organization (WHO) published ‘Standards for Prosthetics and Orthotics’ to improve these services globally. Research that compares services to the standards assists in developing a baseline against which future development can be measured and identifies areas needing improvement.

**Objectives:**

This article aims to describe prosthetic services in Namibia and compare them to the 60 WHO standards.

**Method:**

A mixed-methods exploratory design was employed. Qualitative data were collected through semi-structured interviews with purposively selected participants, including managers (*n* = 2), service providers (*n* = 9) and users (*n* = 16). The data were analysed using content analysis. In the quantitative phase, cross-sectional surveys were administered to managers (*n* = 2), service providers (*n* = 10) and users (*n* = 120). The data were analysed descriptively. Qualitative and quantitative data were triangulated to determine Namibia’s adherence to the standards.

**Results:**

The triangulated data showed adherence to 14 standards, partial adherence to 24 and non-adherence to 22 standards. As per the standards’ requirements, the government directed the provision of prosthetic services, and a range of prosthetic products was provided free of charge at all levels of care. Funding challenges, no national prosthetics committee, and no databases as well as lacklustre support of providers’ careers and professional development, indicated areas of non-adherence.

**Conclusion:**

A systems-based approach, utilising a people-centred conceptual framework, can aid Namibia and similar countries in implementing the standards.

**Contribution:**

This study is the first to provide information on implementing WHO prosthetic standards in an African setting.

## Introduction

**FIGURE 1:**
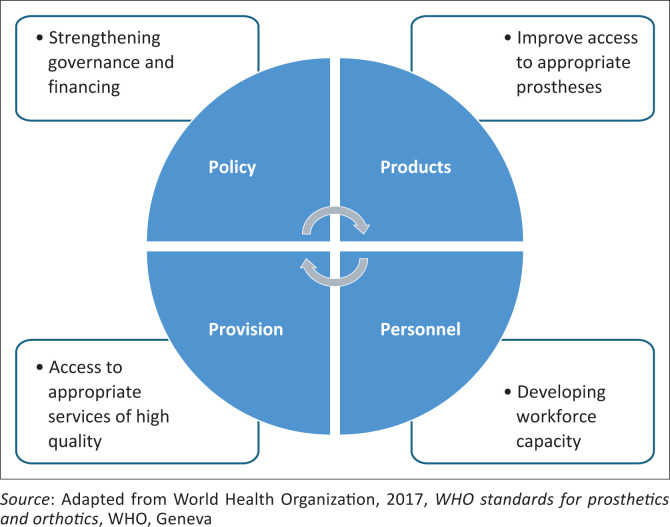
The focus areas of World Health Organization standards for orthotics and prosthetics.

In 2017, the World Health Organization (WHO), in collaboration with the International Society for Prosthetics and Orthotics (ISPO), introduced 60 standards for orthotics and prosthetics (O&P) (WHO 2017). These standards aim to protect the rights of the users by ensuring safe, appropriate, affordable and high-quality prostheses. The standards are organised into four key areas: Policy; Products; Personnel; and Provision (WHO 2017), which together provide a framework for O&P research and service delivery (Figure 1).

These four domains serve as the conceptual framework for this study, which aims to explore the extent to which prosthetic service delivery in Namibia aligns with WHO standards across the domains. To provide context for this exploration, each domain is briefly outlined.

### Policy

The 15 policy standards focus on governance, financing, information collection and sharing. According to the policy standards, governments are responsible for developing, implementing and monitoring national O&P policies. In fulfilling this role, the government should seek guidance from a national committee that includes representatives from all relevant stakeholder groups. Collaboration among stakeholders enhances policy relevance and effectiveness through the integration of diverse perspectives and expertise (MacLachlan et al. 2018; Smith et al. 2022). However, Lang et al. (2019) showed that persons with disabilities are often excluded from policy development and implementation processes in Africa. Furthermore, the absence of policy and poor policy implementation lead to inconsistent and/or protracted decision-making that impedes service delivery (Allen et al. 2020; Baudin et al. 2020).

### Products

The nine (WHO 2017) product standards emphasise the importance of affordable, high-quality and cost-effective products that are appropriate to user needs. This means that a range of components (i.e. prosthetic knees or feet) and materials should be available in a country (WHO 2017). Products must be reliable and should facilitate optimal function and participation in economic, cultural and lifestyle activities (Baudin et al. 2020). They must also be suited to local climate and terrain (Kam et al. 2015). Although a broad spectrum of prosthetic options exists globally, from basic models to advanced technologies, users in many African countries lack access to the latter because of the financial barriers (Wyss et al. 2015). Product selection should be informed by the user’s physical capacity, safety needs and economic circumstances (Asif et al. 2022).

### Personnel

Prostheses are complex devices, custom-made to fit the needs and morphology of individual users. Thus, capable providers with clinical and technical skills are essential to prosthetic service delivery (Baudin et al. 2020). The 12 personnel standards further emphasise the need for adequately trained prosthetists to meet national demand. Standards also call for clearly defined career pathways, ongoing professional development, mentorship, and supervision to maintain quality and ensure the sustainability of services (Baudin et al. 2020).

### Provision

The final domain comprises 23 service provision standards that emphasise integrating prosthetic services into national healthcare systems across all levels of care. Services should be non-discriminative, accessible, user-centred and tailored to meet individual needs (WHO 2017). In many African countries, prosthetic services are concentrated in urban areas, limiting access for rural populations (Ennion & Johannesson 2018; Kam et al. 2015; Urva et al. 2023). Additional barriers such as high transportation costs, poor infrastructure, lack of standard operating procedures, limited government support, and shortages of trained staff and materials further impede service access (Allen et al. 2020; Koho 2024; Urva et al. 2023; Wyss et al. 2015).

### Rationale and aim

According to the WHO (2017), countries can use these standards to develop a baseline for monitoring the progress of prosthetic systems and services. Lemaire, Supan and Ortiz (2018) emphasise the importance of country-wide studies that assess adherence to the standards. No research could be identified on the topic. This lack of research hampers the development of evidence-based policy and service provision, which in turn can negatively impact user outcomes. The current study aimed to describe prosthetic services in Namibia and compare them to the 60 WHO standards. Findings might be relevant to a broader audience because of the scarcity of similar work.

### Context

Namibia is located on the west coast of Southern Africa, covering an area of 825 000 square kilometres and has a population of 2.5 million (World Bank 2024). Despite ongoing urbanisation, 60% of the population resides in rural areas. Namibia faces notable socio-economic disparities as an upper-middle-income country with a Gross Domestic profit (GDP) of $5031.10 per capita (World Bank 2024). Income and asset inequality remain significant, with a Gini index of 59.1 (World Bank 2024).

Namibia operates a two-tiered health system, with over 80% of the population relying on public healthcare services (Christians 2020). The population’s geographical dispersion, long distances, challenging terrain and climate conditions pose logistical challenges for service delivery (Eide et al. 2015). The Ministry of Health and Social Services (MOHSS) offers lower limb prosthetics services through three facilities and mobile outreach clinics.

## Research methods and design

### Design

The study employed a sequential, mixed-methods, exploratory design (O’Leary 2021). A quantitative cross-sectional survey followed a qualitative descriptive study (Bradshaw, Atkinson & Doody 2017). A sequential mixed-methods exploratory design is appropriate when little or no research has been previously conducted on a topic (Kroll, Neri & Miller 2005), as is the case in the present study. Participants’ understanding and narrative description of the situation regarding prosthetic service delivery in Namibia were utilised to provide background on the issue and develop a survey through which results could be quantified.

### Study setting

Participants were recruited from the O&P centres at Windhoek Central Hospital and Oshakati Intermediate Hospital. Windhoek Central Hospital provided around 200 lower limb prostheses per year. The facility employed two orthotists and/or prosthetists, nine orthopaedic technologists, five orthopaedic assistants and three support staff. Oshakati delivered around 120 lower limb prostheses per year. The workforce consisted of one orthotist and/or prosthetist, five orthopaedic technologists, two orthopaedic assistants and three support staff members.

### Population, sampling and participants

Twenty-seven individuals (2 managers, 9 service providers and 16 users) participated in the qualitative descriptive study. The 132 participants in the quantitative phase included the same 2 service managers, 10 service providers and 120 service users.

Participants were identified and/or sampled in the following manner:

- The 2 service managers from the two settings participated in both phases.
- Out of a possible 22 service providers, 9 were purposively sampled for the qualitative phase, while 10 completed the survey. They were included if they had 5 or more years of relevant work experience.
- The population of prosthetic users was 691 who received prosthetic services at one of the two O&P centres or their outreach points between 2013 and 2018. Users had to be at least 18 years old and free from additional impairments that might hinder prosthetic function. The second author used purposive sampling to select 16 users to participate in the qualitative phase. During sampling, users from both facilities were included. To ensure variation among participants, both men and women, of different ages, with different amputation levels, and from rural and urban areas were selected (Palinkas et al. 2016).
- The sample size calculation indicated that 376 users would provide a 95% confidence level (95% confidence interval). Unfortunately, the actual sample was much smaller because of the practical difficulties, such as users having passed away or not meeting the study criteria because of additional health conditions. Furthermore, incomplete records, relocation and changed phone numbers made it challenging to contact prosthetic users. This reduced access to potential participants prevented random sampling. Therefore, convenience and snowball sampling strategies were used. In this manner, 120 users were identified. Both the non-probability sampling strategies and the reduced number of participants hampered the validity of the results.

### Data collection tools and methods

Three interview schedules, one for each participant group, were developed in-house based on the WHO standards for prosthetics and orthotics (WHO 2017). The second author conducted one-on-one face-to-face interviews. In instances where there was language barrier, an interpreter assisted with data collection. Quantitative data were collected using two self-developed surveys: one for managers and providers, and one for the users. These surveys were based on qualitative findings and the WHO standards for prosthetics and orthotics (WHO 2017).

### Data analysis

Qualitative data were analysed through content analysis, as it allowed for some quantification of the data (Vaismoradi et al. 2016). Data analysis was deductive, focusing on adherence or non-adherence to the 60 standards. Quantitative data were analysed with Statistical package for the Social Sciences (SSPS) version 28.0. Continuous data were presented through means and standard deviations. Nominal categorical data were presented through frequencies and percentages. Counting response frequencies facilitates an understanding of the data distribution. Data from the two phases were triangulated, i.e. narrative information from interviews was used to contextualise and add explanations to numerical information from the survey on whether a specific standard was adhered to or not. The results are presented according to the extent to which standards were adhered to rather than themes, as per the article’s aim.

### Rigour

Data from the different methods, tools and sources were analysed separately, triangulated and presented in an integrated manner (O’Leary 2021) to provide a holistic picture and enhance rigour. Trustworthiness of the qualitative phase was further supported through purposive sampling, seeking data saturation, using tried data collection and analysis methods, peer debriefing, and describing the methods, setting and participant demographics. The surveys were not formally tested for criterion validity and reliability; however, input was sought from prosthetists and academics, and a pilot study was conducted to enhance content validity and face validity.

### Ethical considerations

Ethical approval was obtained from Stellenbosch University Health Research Ethics Committee on 17 December 2020 (Ref No. S20/04/090), and permission to collect data was obtained from the Research Committee of the Ministry of Health and Social Services in Namibia (Ref No. 17/3/3 CML). Participation was voluntary, and all participants provided written informed consent. Data are saved on password-protected computers and in cloud locations. Only the authors have access to the data.

## Results

### Demographics

The ages of service providers and managers ranged from 39 to 46 years, with a mean age of 42.33 years (s.d. = 2.71). Their work experience varied between 11 and 21 years. Among them, three were prosthetists and orthotists, and nine were orthopaedic technologists (Table 1 & Table 2). Of the 120 user participants, the majority were male (74.2%, *n* = 89) and from the Khomas region (65%, *n* = 78). Their mean age was 48.72 years (s.d. = 2.71), and they had been using a prosthesis for an average of 16.30 years (s.d. = 12.25) (Table 2).

**TABLE 1:** Qualitative phase demographic information.

Participant number	Gender	Age (years)	Region	Work experience (years)	Professional category	Prostheses use (years)	Level of amputation	Distance from the facility (km)
Manager 01	M	46	Khomas	19	Orthotist and Prosthetist	-	-	-
Manager 02	M	45	Oshana	20	Orthotist and Prosthetist	-	-	-
Provider 01	M	43	Khomas	19	Orthotist and Prosthetist	-	-	-
Provider 02	M	40	Khomas	18	Orthopaedic Technologist	-	-	-
Provider 03	F	39	Khomas	18	Orthopaedic Technologist	-	-	-
Provider 04	M	39	Khomas	11	Orthopaedic Technologist	-	-	-
Provider 05	M	40	Khomas	11	Orthopaedic Technologist	-	-	-
Provider 06	M	39	Oshana	12	Orthopaedic Technologist	-	-	-
Provider 07	F	43	Oshana	19	Orthopaedic Technologist	-	-	-
Provider 08	M	42	Oshana	18	Orthopaedic Technologist	-	-	-
Provider 09	M	44	Oshana	19	Orthopaedic Technologist	-	-	-
User 01	M	81	Khomas	-	-	41	TT	5
User 02	F	36	Khomas	-	-	15	TT	4
User 03	M	38	Khomas	-	-	31	TT	498
User 04	F	60	Khomas	-	-	18	TT	498
User 05	M	58	Khomas	-	-	5	TF	268
User 06	F	52	Khomas	-	-	4	TF	268
User 07	F	64	Khomas	-	-	48	TT	268
User 08	F	63	Khomas	-	-	11	TT	361
User 09	F	37	Khomas	-	-	30	TF	338
User10	F	34	Khomas	-	-	24	TF	317
User 11	M	55	Oshana	-	-	4	TF	3
User 12	M	31	Oshana	-	-	28	TT	4
User 13	F	71	Oshana	-	-	41	TT	92
User 14	F	60	Oshana	-	-	28	TT	92
User 15	M	60	Oshana	-	-	36	TT	205
User 16	M	59	Oshana	-	-	32	TT	205

M, male; F, female; TT, transtibial; TF, transfemoral.

**TABLE 2:** Quantitative phase demographic information.

Participants	*n*	Demographic variables	Min	Max	Range	Mean	s.d.
Managers and providers	12	Age	39	46	7	42.33	2.708
		Years of experience	11	21	10	17.67	3.257
Users	120	Age	19	88	69	48.72	15.965
		Years since amputation	4	50	46	18.65	12.965
		Years of using a prosthetic	3	44	41	16.30	12.246
		Age of current prosthesis (years)	1	36	35	5.31	6.073
		Distance to nearest prosthetics facility (km)	1	1229	1228	258.38	265.610

Min, minimum; Max, maximum; s.d., standard deviation.

### Adherence to World Health Organization standards

**FIGURE 2:**
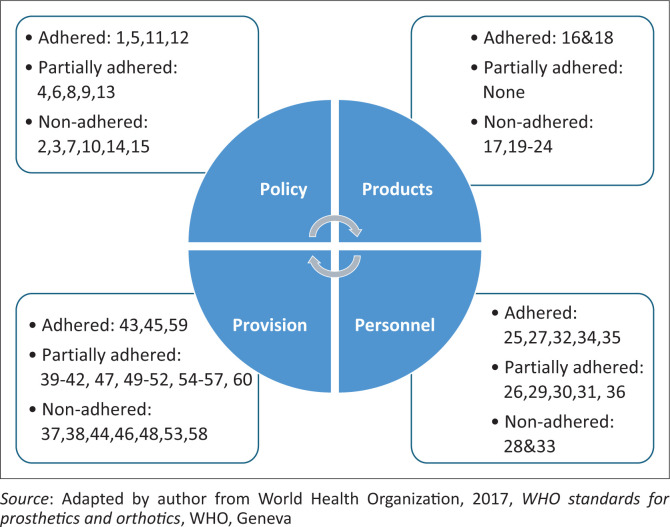
Adherence in Namibia to World Health Organization standards for orthotics and prosthetics.

Figure 2 shows that, across the four areas, 14 (23%) of the 60 standards were adhered to, 24 (40%) were partially adhered to, and 22 (37%) were not adhered to at all.

### Standards that were adhered to

The Namibian Government, through the Ministry of Health and Social Services, guided and regulated prosthetic service provision in the country (standard [Std.] 1, 5) through strategic and operational plans (MOHSS, 2018) (Std. 59):

‘When it comes to long-term operational plans and performance indicators, we do not work on our own, we are part of the Ministry and we contribute to the objectives of the Ministry, so we are using the annual plans for the Ministry and we are also using the strategic plan, which is a long-term plan of the Ministry …’ (Service Manager 02)

The country did not have universal healthcare, but government prosthetic services were provided free of charge to users (Std. 11), and prosthetic services were included in what little insurance systems the country had (Std. 12).

A wide range of contextually appropriate lower limb prosthetics products (Std. 16), classified according to international standards (Std. 18), were available (Table 3).

**TABLE 3:** Suspension systems and prosthetic component availability (*n* = 12).

Variable	Components	Yes
Suspension systems	Cuffs, Straps, and Belts	10
	Pin and lock	11
	Suction without a liner	5
	Suction with liner	8
	Vacuum assisted	7
	Self-suspending socket	10
Prosthetic: Knees	Manual locking	9
	Single axis	9
	Weight activated	10
	Polycentric	11
	Hydraulic	2
	Pneumatic	8
	Micro-processor	0
Prosthetic: Feet	Solid ankle cushion heel	12
	Dynamic	12
	Multi-axis	1
	Flexible Keel	4
	Micro-processor	0

More expensive products were not always provided because of budgetary constraints:

‘…[*W*]hat we are providing meets the needs of the patients but at a certain level, you always have one or two who go an extra mile with their needs, like the blades … our prosthetics products can ferry our patients to where they are working and where they are staying … we cannot satisfy everyone but satisfying 95 to 98% of clients.’ (Service Provider 04)

‘He is saying, he is very much happy … the prosthetic leg is helping him a lot, and even if we were in a jungle and probably the lion was approaching us, he might even run faster than any of us, and he might leave us behind.’ (Service User 15, Through an Interpreter)

Most users (85.9%; 103) indicated that the prosthetic components they received addressed their functional needs (Table 4). However, the prosthesis affected the ability to work of 40.8% (*n* = 49) of users. Furthermore, 50.8% (*n* = 61) of users strongly agreed that they depend on others more than they would like to.

**TABLE 4:** Users’ experience of prosthetic functioning and services (*n* = 120).

Function and services	Strongly disagree		Disagree		Agree		Strongly agree		Not applicable	
	*n*	%	*n*	%	*n*	%	*n*	%	*n*	%
Meet functional needs	2	1.7	15	12.5	35	29.2	68	56.7	-	-
Interferes with the ability to work	8	6.7	18	15.0	45	37.5	49	40.8	-	-
Limited in types of work	10	8.3	41	34.2	34	28.3	35	29.2	-	-
To be dependent on others	2	1.7	21	17.5	36	30.0	61	50.8	-	-
Satisfied with maintenance and repair	1	0.9	11	9.2	28	23.3	63	52.4	17	14.2
Involved in planning and goal setting	4	3.3	30	25.0	42	35.0	44	36.7	-	-
Satisfied with planning and goal setting	2	1.7	31	25.7	38	32.0	49	41.0	-	-

Maintenance and repair of prosthetic products were part of the provided services (Std. 43):

‘The maintenance and repair, we look at the cosmetic finishing of the prosthesis, we look at the replacement of components … at the socket replacement in cases when the patient outgrows the prosthesis or the stump has reduced in volume. So, we do everything.’ (Service Provider 04)

About 85.8% (*n* = 103) of users took their devices for repair or maintenance in the past, and 75.83% (91) were satisfied with the repair and maintenance they received (Table 4).

The state regulated service providers through the Health Professions Council of Namibia (Std. 34). Professionals were adequately qualified (Std. 25), and their training met international standards (Std. 27). Every service delivery site had at least one prosthetist and/or orthotist (Std. 32) who provided supervision to and was responsible for services provided by technicians and other non-clinical staff (Std. 35). Participants indicated that more prosthetists and orthotists were needed:

‘There is only one position for a prosthetist and orthotist at this facility. This person is expected to supervise and guide all the clinical and technical duties, it’s a situation that stretches someone to an extent that sometimes quantity outweighs the quality and then the professional guidance is a bit compromised, so, it is a problem.’ (Service Manager 02)

### Partially adhered to standards

The national guiding framework for prosthetics service provision (MOHSS 2001) (Std. 4) was outdated:

‘They were formulated more than 10 years ago … They are not useful because things have changed, as there is a change in technology and new international policies.’ (Service Provider 01)

To be specific, 95% (114) of users were not aware of any policy.

The MOHSS monitored prosthetic services (Std. 6). However, the most recent annual report for the 2017–18 financial year [20] had figures for Windhoek Central Hospital only.

Participants felt the budget was insufficient:

‘…[*Y*]ou might budget a certain amount and at times you might get what you budgeted for but many a time we do get less or not getting anything at all, and sometimes the money is only made available towards the end of the year.’ (Service Provider 08)

They indicated that an increase of 4–10 million Namibian dollars is needed. This might indicate that the cost of prosthetic services is not assessed periodically, or if assessed, budgets are not adjusted accordingly (Std. 9). Financial shortfalls hamper service delivery.

‘We have patients that are coming in with broken prostheses and we still send them back home because we do not have the materials … some of them their prostheses are in very bad shape and they are in dire need of these services but at the moment we are not able to provide anything because there are no materials … We have a challenge with the issue of equipment maintenance and replacement because of the funds; sometimes, there is no budget at some point to replace certain equipment.’ (Service Provider 04)

This also causes shortfalls in materials and consumables:

‘They usually don’t have materials, every time she goes there, they tell her the same story of no materials.’ (Service User 07; Through interpreter)

Budgets were not ring-fenced:

‘…[*W*]hen there is a crisis or other needs of the ministry, these services usually take a back seat because there is a narrative that prosthetic patients’ “don’t die,” therefore some of the priorities of these services end up being overlooked.’ (Service Provider 02)

International support was limited to the training of professionals, and it was unclear whether this contributed to the long-term aims of strategic plans and whether it was in alignment with national health plans (Std. 8). Continuous Professional Development (CPD) was compulsory but poorly enforced (Std. 30):

‘I have seen a document that talks about CPD, but the reality is that it’s not reinforced to a point where one is required to submit the points accumulated.’ (Service Provider 09).

Consultations between prosthetists and other rehabilitation professionals were irregular with limited multidisciplinary teamwork (Std. 26). Other rehabilitation professionals were regarded as having fair knowledge about prosthetics (Std. 29). Most managers and providers indicated that they occasionally provided training to other healthcare team members (Std. 50).

Service providers were unhappy with the existing career structure (Std. 36):

‘…[*I*]t is a government-driven service, so it is more of service delivery than career development…’ (Service Provider 02)

First-time users could not choose either a provider or products (Std. 39). On return visits, users were often allowed to consult with a service provider of their choice (Std. 39):

‘…[*F*]or the first time, they do not have any choice. But for the second time, they can always select, that I want to be assisted by the person who assisted me last time I was here.’ (Service Provider 03)

‘Clients do not have a choice on what is to be used on their prostheses, it is us professionals who choose what we are going to provide to them … we are the ones who do the assessment, and we know what is right for the patients because some may opt to go for expensive items, but such a person may end up not using it and just keep it under the bed and since it is expensive somebody else may need it.’ (Service Provider 03)

Another provider indicated that with empowerment, users could choose components:

‘We always discuss with the patients, we give them options and we show them some pictures and samples that we have, then they are allowed to choose … we still have to consider the type of components available, then they could choose from those.’ (Service Provider 05)

Users concurred that with experience, they took a more active role and provided input in decisions regarding their prosthetic components:

‘The very first prosthesis that she got, she was not allowed to choose the type of leg that she wanted or the materials that were used, she was just informed on the type of leg that she was going to get. But then the last one that she got, she chose the type of leg and materials that she wanted.’ (Service User 04, through an interpreter)

They also participated in setting goals and planning treatments (Std. 52):

‘I think, for every step, we try very much to involve the service user and the caregivers, because at the end of the day, if I make my own decision that does not sit well with the user, it means the user might not use the product, so we try by all means to involve them as much as possible.’ (Service Provider 06)

However, further explanations reveal an emphasis on education rather than collaboration:

‘From the beginning when we are doing the evaluation, we give them a plan on how we are going to provide them with the device and then we tell how the device should be taken care of and then we provide with information of how they supposed to be coming back to use and everything needed. So, we believe that such an arrangement gives them an overview on how to use the device and what they are supposed to do for the device to have a long-life span.’ (Service Provider 02)

Most users, 71.5% (*n* = 86), were involved during assessment, planning and goal setting. Most were also satisfied (72.5%, *n* = 87) with the goals and treatment plan (Table 2).

Prosthetic services were available to everyone, without any discrimination, at all three levels of care (albeit through outreach clinics at the primary level) and supported through referral pathways (Std. 40 & 42). Long distances of up to 1229 kilometres (mean 258.38; s.d. 265.61) and transport challenges made access difficult for some. Only 32.5% (*n* = 39) of users utilised free government transport. Users experienced discrimination when accessing government transportation in what appears to be an informal triage process:

‘They [patients] do complain that they are always the last group when it comes to boarding the hospital’s official bus because they are seen that their cases are not considered as an emergency, therefore, they can wait, so other groups of patients are the ones prioritised first.’ (Service Provider 05)

Thus, while prosthetic services were part of the healthcare sector (Std. 41), it might be deemed of lesser importance than other healthcare services.

Facilities were mostly accessible and safe (Std. 47):

‘She is saying the building is built in a friendly manner … and persons with artificial legs could go in without any difficulties in accessing it … the signs are there to read but for someone who has not been there, some people can always direct you and it is very easy to access through directions.’ (Service User 08)

Concerns were raised (3/16) that directions were not always visible:

‘She says the direction signs from the main entrance of Windhoek Central Hospital are not visible and therefore it is not easy to tell where the orthopaedic facility is.’ (Service User 02, through an interpreter)

The manufacturers’ instructions were usually followed. Deviations were not documented (Std. 54):

‘Yes, we try to follow the component manufacturers’ guidelines, but sometimes we end up mixing. For example, a certain knee joint is supposed to be matched with a certain foot of a certain K level, but at the end of the day we end up using a K4 or K3 knee joint with a K2 foot because maybe at that moment, we do not have a suitable foot …. because maybe it is very expensive, therefore we end up buying, for example, a SACH foot which is a K1, where this will result in the inappropriate matching of components or mixing wrong components of different activity levels.’ (Service Provider 01)

About 98.3% (*n* = 118) of users received training that enabled effective use of the prostheses (Std. 55), but training was focused on indoor mobility and sometimes limited by transport challenges:

‘He was trained here at this facility on how to walk, and that really helped him, and first he was not confident enough, but as training days moved on, he developed confidence. It lasted 2 weeks, and then he went home after that, and he trained himself on how to walk in the sandy areas because here the surface is nicely flat, but at home there is uneven ground, and that is why he needed to train more.’ (Service User 11, through an interpreter)

Users had the final say about the fit and functionality of the prosthesis (Std. 56), as indicated by 88.3% (*n* = 106) of users in the survey and through qualitative data:

‘That decision is for the patient, there is no way you can impose a prosthesis on someone who does not want it.’ (Service Provider 04)

‘For me, usually I do not leave if I am not happy with the work done, so I usually stay up until I am happy with everything, then I go.’ (Service User 09)

Follow-up was done on an informal basis (Std. 58):

‘…[*T*]he follow-ups we have, we normally just inform our patients that if the device is giving them problems, they can always come back, but we don’t give them a time frame on when they are supposed to come back unless there is a specific thing that we are looking at.’ (Service Provider 02)

This manner of follow-up suited users who thought it unnecessary to return to the centre if they did not experience problems with the prosthetic:

‘She is saying the kind of follow-up that she got, she was told to come back only when there was a problem, so they never gave her a time frame, and then she went back when she encountered a problem with her leg and the service was smooth … it is more important if one goes back only when there is a problem and says I have this problem, that way it is much easier.’ (Service User 08)

Other users felt regular follow-ups could be helpful as they might be unaware of a problem with the prosthesis:

‘At the moment, some patients who have problems with their prosthetic legs do not even know what is wrong, so it would be good if the professionals could always make follow-ups.’ (Service User 06)

### Standards not adhered to

Users were not included in policy development, planning, implementation, monitoring and evaluation of services (Std. 2, 38). There were no national prosthetic committees or databases (Std. 3, 14). Neither was there a national priority prosthetics products list (Std. 17). Since very little information was documented and even less research was conducted (Std. 23), international collaboration and sharing of experiences, data and research (Std. 7) will be challenging. The economic benefits of prosthetic services were not analysed (Std. 10).

Awareness-raising strategies for prosthetic services were lacking (Std. 15):

‘…[*T*]here is not much awareness about these services that we offer, and very few people are aware of these services. That means not everyone who is amputated will come to the facility, so only part of them are getting the service.’ (Service Provider 01)

No professional prosthetic training was provided in Namibia at the time of the study (Std. 28). Strategies for staff retention were not in place (Std. 33).

Without equipment to examine the quality of devices, quality was equated with the lifespan of the limb (Std. 22). Prosthetic components and materials were not subject to regulation. Components were reused without quality control testing (Std. 20, 21). Even so, lower limb prosthetic components were commonly reused. No prosthetic components were manufactured locally (Std. 24), and neither were prosthetic tools, consumables, machines and components exempt from import duty (Std. 19).

Facilities had no equipment maintenance, repair and replacement plans (Std. 48), and little funding was allocated for equipment maintenance and replacement. Prosthetic manufacturing equipment is specialised and could not be repaired by the hospital maintenance crew:

‘…[*W*]e don’t have technicians to maintain the machines. So, you will find that we have a lot of machines in the department, but most of them are not working and there is no one to maintain them.’ (Service Provider 07)

User outcomes were informally evaluated and not documented (Std. 57):

‘Most of the time we don’t ask how the first one or the old one and to know whether it was good or something, I think we have neglected that part …. It’s very important to get such feedback, which we are neglecting.’ (Service Provider 07)

No formal peer support or counselling was provided (Std. 53). Users found informal peer counselling valuable:

‘If there is a new patient under training and there is an old user who probably came in for some reason, maybe for replacement or repair of his device, we can then ask him to go and assist or share his experience with the new user, that is the only platform that they can use. We usually leave them discussing while they are at our facility, then the old user will provide hints to the new user on how to use the device. But we do not have an official platform where we call in the old users to come and guide the new users.’ (Service Provider 02)

‘The first time before she received her prosthesis, the patients that she found there actually explained more to her about how to use the prosthesis, and she felt that she was helped, it boosted her confidence, and it made it easier for her to accept her new leg.’ (Service User 04, through an interpreter)

Finally, disaster preparedness was not part of prosthetic service plans (Std. 44). No documented policy that outlines patient-centred services and safeguards the rights of prosthetic service users could be identified (Std. 37). The probability of integrating prosthetic services with broader assistive product services had not been investigated (Std. 46).

## Discussion

The Namibian government developed, coordinated and provided prosthetic services through the MOHSS. However, the government can provide additional practical guidance and support to enable adherence to all 60 standards. An outdated national guiding document MOHSS 2001 may lead to disregarding current evidence, resulting in services that do not respond optimally to the needs of users, providers and the context (Baudin et al. 2020). Additionally, users were unaware of the policy and were not involved in its development, implementation or monitoring. This can leave them disempowered as they might not fully know the available options and their rights (Alemu, Girma & Mulugeta 2021; MacLachlan et al. 2018). A national prosthetics committee can provide a platform where all voices, including those of users, can be heard (MacLachlan et al. 2018). Peta (2017) and Trafford and Swartz (2023) presented ways in which Africans with disabilities can be involved in policy processes to ensure that their opinions are included.

A reasonably wide range of context-appropriate, high-quality prosthetic products was available free of charge in Namibia. This heartening finding was remarkable as free-of-charge prosthetic provision often results in the use of entry-level products (Pienaar & Visagie 2019). As in other African settings, rural users experienced access problems due to distance and transport barriers (Ennion & Johannesson 2018; Kam et al. 2015; Urva et al. 2023). The Namibian healthcare budget accounts for 14.5% of total government spending, one of the highest rates in Africa (World Bank 2024). However, what percentage of this budget is earmarked for lower limb prosthetic services remains unknown. What was found was that the funding was insufficient and was not always utilised as per plans. Insufficient funding can deprive persons with amputation of their right to a prosthesis with negative functional, economic and quality of life effects (Cote 2021).

The absence of a database that captures information on amputations and prosthetic services means that service planning and resource allocation are conducted with limited evidence-based guidance. This can lead to inequitable service access (Akram et al. 2018). The information deficit started at the service delivery level with poor documentation of treatment interventions and outcomes and contact details, as the struggle to identify study participants showed. Clinical records are legal documents and should be maintained. They can also form the basis for a national database. In addition to service planning, a database can stimulate research and facilitate national and international information sharing and collaboration. Collaboration kindles ideas and interventions while saving costs as repetition and outdated, ineffective strategies are pointed out (Ramstrand et al. 2020).

Exemption from import duty will be cheaper than funding and running a manufacturing plant. Importing components gives users access to a large range of products and a bigger choice than what local manufacturing can provide. However, for user safety and to prevent wasting of funds, an independent body must regulate prosthetic components and materials, both new and recycled, to ensure each component complies with the international organisation for standardisation (ISO) or equivalent standards. Similarly, government tender contracts must include a clause that prevents purchasing components that are not ISO certified. Structural testing of prosthetic products (both new and second-hand) protects users from harm resulting from component failure. Substandard products require frequent repairs and replacement, making them uneconomical.

For every million people, there should be between 5 and 10 prosthetists (WHO 2017). Thus, Namibia should have 10 to 20 prosthetists, rather than the current 3, for its population of 2.5 million. A shortage of prosthetists is common in Africa, attributed to the scarcity of institutions offering accredited prosthetic training courses in the region (Aduayom-Ahego, Ehara & Anareme 2017). The challenges of adhering to CPD can likely also be attributed to the limited number of local training institutions (Aduayom-Ahego et al. 2017) and the scarcity of locally based product suppliers. Another African research has also found limited continuing professional development among prosthetists (Aduayom-Ahego et al. 2017; Magnusson, Shangali & Ahlström 2016; McDonald, Kartin & Morgan 2020). Not staying abreast with new evidence and developments negatively impacts service quality (Highsmith 2015).

Staff development, retention and career pathways are critical components of an adequate and competent workforce. Magnusson and Ahlström (2012) showed that prosthetists in Sierra Leone were not happy with their remuneration. Low salaries can demotivate service providers and make retaining and appointing staff difficult. Mwetulundila (2019) found that gender and cultural affiliations caused barriers to career progression in nursing in Namibia. The present study found no evidence in this regard.

Client-centred services require people to have freedom of expression and choice (Langberg, Dyhr & Davidsen 2019). Users must be allowed to choose service providers and prosthetic components (O’Keeffe & Rout 2019). Providers are responsible for empowering users to make informed choices (Farrar, Niraula & Pryor 2022; Langberg et al. 2019). If service users are not part of the decision process regarding prosthetic components, they might be unhappy with the prosthesis and abandon it (Anderson et al. 2022). First-time or inexperienced users can also learn from experienced users through peer support. Peer support may assist them physically and emotionally, and build their confidence to decide which components are appropriate to their needs (Highsmith 2015).

Poor fitting, discomfort and functional challenges can be identified and rectified during follow-up appointments before secondary complications such as blisters develop or the user abandons the devices (Lee & Veneri 2018). First-time users should be seen within 3 weeks after fitting (O’Keeffe & Rout 2019), with further regular appointments every 6–12 months.

One-stop facility that has a variety of assistive products available in one place and offers assessment and interventions by a multidisciplinary team should be considered because persons using prostheses often need additional devices such as crutches or a wheelchair (Smith et al. 2018). One-stop facilities enhance the accessibility of assistive products, thus saving users’ time and money (WHO 2022). However, the specific nature of such facilities in Namibia and Africa must be further explored as the Norwegian model (WHO 2022) may be unsuited to settings with vast rural areas and highly dispersed populations. Assistive technology (AT) models in Africa must suit the geographical structures of different countries and consider dispersed populations and long travel times in often harsh environments (Ennion & Johannesson 2018; Kam et al. 2015; Wyss et al. 2015).

Poorly functioning or malfunctioning manufacturing machines and tools can cause delays in service delivery and result in prolonged waiting times for prostheses. Thus, a maintenance plan must be in place. Furthermore, skilled technicians who can do maintenance and repair must be trained, employed and supported with funding for parts. To reduce costs, one or two people can be trained and employed to travel between facilities. This is an area where international collaboration with industry might be beneficial.

### Limitations

A significant limitation of this study was the relatively small number of participants in the quantitative phase, along with the use of non-random sampling, which may have affected the applicability of the findings (O’Leary 2021). Additionally, the lack of demographic and amputation-related data of individuals who could not be traced limited our ability to determine whether these individuals differed in important ways from the study participants. Moreover, the surveys used in this research were not formally tested for validity or reliability, which may have affected the strength of the results.

## Conclusion

Out of the 60 standards, 14 have been fully implemented. Of the remaining 46, some require complex interventions and substantial financial investment for effective implementation. It is recommended that the budget allocated to prosthetic services be increased and ring-fenced to ensure long-term sustainability. Furthermore, initiatives should be undertaken to establish clear career pathways within the profession and to facilitate access to CPD opportunities, both locally and internationally.

Several standards, however, can be addressed with minimal additional expenditure. These include fostering user participation in the planning, monitoring and evaluating services; establishing a representative prosthetics committee; systematically recording and analysing service-level data for integration into a national database; developing a national prosthetic product priority list; and formalising peer support programmes for users.

A systems-based approach, guided by the 10 Ps people-centred conceptual framework developed by MacLachlan and Scherer (2018), is recommended as an effective model for prosthetic service delivery in Namibia and other comparable countries. With the support of the ISPO, it is feasible for more countries to conduct nationwide research to assess how prosthetic services can be enhanced to align with the WHO standards.

## References

[CIT0001] Aduayom-Ahego, A., Ehara, Y. & Anareme, K., 2017, ‘Prosthetics and orthotics education in sub-Saharan Africa: Issues and challenges’, *Ec Orthopaedics* 6(2), 81.

[CIT0002] Akram, M.A., Mahmud, M.I., Ashraf, S.R.B., Awal, S. & Talapatra, S., 2018, ‘Enhancing the healthcare service using quality function deployment and database management system in the outpatient department of a government hospital of Bangladesh’, *International Research Journal of Engineering and Technology* 5(4), 2022–2029.

[CIT0003] Alemu, W., Girma, E. & Mulugeta, T., 2021, ‘Patient awareness and role in attaining healthcare quality: A qualitative, exploratory study’, *International Journal of Africa Nursing Sciences* 14, 100278. 10.1016/j.ijans.2021.100278

[CIT0004] Allen, A.P.T., Bolton, W.S., Jalloh, M.B., Halpin, S.J., Jayne, D.G. & Scott, J.D.A., 2020, ‘Barriers to accessing and providing rehabilitation after a lower limb amputation in Sierra Leone – A multidisciplinary patient and service provider perspective’, *Disability and Rehabilitation* 44(11), 2392–2399. 10.1080/09638288.2020.1836043 33261506

[CIT0005] Anderson, C.B., Kittelson, A.J., Wurdeman, S.R., Miller, M.J., Stoneback, J.W., Christiansen, C.L. et al., 2022, ‘Understanding decision-making in prosthetic rehabilitation by prosthetists and people with lower limb amputation: A qualitative study’, *Disability and Rehabilitation* 45(4), 723–732. 10.1080/09638288.2022.2037745 35389313 PMC9537359

[CIT0006] Asif, M., Tiwana, M.I., Khan, U.S., Qureshi, W.S., Iqbal, J., Rashid, N. et al., 2022, ‘Advancements, trends and future prospects of lower limb prosthesis’, *IEEE Access* 9, 85956–85977. 10.1109/ACCESS.2021.3086807

[CIT0007] Baudin, K., Mullersdorf, M., Sundstrom, A. & Gustafsson, C., 2020, ‘The policies of provision of assistive and welfare technology – A literature review’, *Societies* 10(1), 22. 10.3390/soc10010022

[CIT0008] Bradshaw, C., Atkinson, S. & Doody, O., 2017, ‘Employing a qualitative description approach in health care research’, *Global Qualitative Nursing Research* 4, 2333393617742282. 10.1177/2333393617742282 29204457 PMC5703087

[CIT0009] Christians, F., 2020, ‘Country profile – Primary healthcare and family medicine in Namibia’, *African Journal of Primary Health Care and Family Medicine* 12(1), e1–e3. 10.4102/phcfm.v12i1.2242 PMC7061223 32129644

[CIT0010] Cote, A., 2021, ‘Social protection and access to assistive technology in low-and middle-income countries’, *Assistive Technology* 33(Suppl. 1), 102–108. 10.1080/10400435.2021.1994052 34951824

[CIT0011] Eide, A.H., Mannan, H., Khogali, M., Van Rooy, G., Swartz, L., Munthali, A. et al., 2015, ‘Perceived barriers for accessing health services among individuals with disability in four African countries’, *PLoS One* 10(5), e0125915. 10.1371/journal.pone.0125915 25993307 PMC4489521

[CIT0012] Ennion, L. & Johannesson, A., 2018, ‘A qualitative study of the challenges of providing pre-prosthetic rehabilitation in rural South Africa’, *Prosthetics and Orthotics International* 42(2), 179–186. 10.1177/0309364617698520 28318387

[CIT0013] Farrar, M., Niraula, Y.R. & Pryor, W., 2022, ‘Improving access to prosthetic services in Western Nepal: A local stakeholder perspective’, *Disability and Rehabilitation* 45(7), 1229–1238. 10.1080/09638288.2022.2057599 35387522

[CIT0014] Highsmith, M.J., 2015, ‘Is orthotics and prosthetics a profession?’, *Journal of Prosthetics and Orthotics* 27(4), 115–117. 10.1097/JPO.0000000000000078

[CIT0015] Kam, S., Kent, M., Khodaverdian, A., Daiter, L., Njelesani, J., Cameron, D. et al., 2015, ‘The influence of environmental and personal factors on participation of lower-limb prosthetic users in low-income countries: Prosthetists’ perspectives’, *Disability and Rehabilitation: Assistive Technology* 10(3), 245–251. 10.3109/17483107.2014.905643 24694038

[CIT0016] Koho, M.K., 2024, ‘Enhancing prosthetics and orthotics service delivery in Ghana: A call for standardization, capacity building, and systemic reform’, *Convergence Chronicles* 5(2), 15–27. 10.53075/Ijmsirq/655464355

[CIT0017] Kroll, T., Neri, M.T. & Miller, K., 2005, ‘Using mixed methods in disability and rehabilitation research’, *Rehabilitation Nursing Journal* 30(3), 106–113. 10.1002/j.2048-7940.2005.tb00372.x 15912675

[CIT0018] Lang, R., Schneider, M., Kett, M., Cole, E. & Groce, N., 2019, ‘Policy development: An analysis of disability inclusion in a selection of African Union policies’, *Development Policy Review* 37(2), 155–175. 10.1111/dpr.12323

[CIT0019] Langberg, E.M., Dyhr, L. & Davidsen, A.S., 2019, ‘Development of the concept of patient-centredness – A systematic review’, *Patient Education and Counselling* 102(7), 1228–1236. 10.1016/j.pec.2019.02.023 30846206

[CIT0020] Lee, D.J. & Veneri, D.A., 2018, ‘Development and acceptability testing of decision trees for self-management of prosthetic socket fit in adults with lower limb amputation’, *Disability and Rehabilitation* 40(9), 1066–1071. 10.1080/09638288.2017.1286694 28637137

[CIT0021] Lemaire, E.D., Supan, T.J. & Ortiz, M., 2018, ‘Global standards for prosthetics and orthotics’, *Canadian Prosthetics & Orthotics Journal* 1(2), 1–5. 10.33137/cpoj.v1i2.31371

[CIT0022] MacLachlan, M., Banes, D., Bell, D., Borg, J., Donnelly, B., Fembek, M. et al., 2018, ‘Assistive technology policy: A position paper from the first global research, innovation, and education on assistive technology (GREAT) summit’, *Disability and Rehabilitation: Assistive Technology* 13(5), 454–466. 10.1080/17483107.2018.1468496 29790393

[CIT0023] MacLachlan, M. & Scherer, M.J., 2018, ‘Systems thinking for assistive technology: A commentary on the GREAT summit’, *Disability and Rehabilitation: Assistive Technology* 13(5), 492–496. 10.1080/17483107.2018.1472306 29772950

[CIT0024] Magnusson, L. & Ahlström, G., 2012, ‘Experiences of providing prosthetic and orthotic services in Sierra Leone – The local staff’s perspective’, *Disability and Rehabilitation* 34(24), 2111–2118. 10.3109/09638288.2012.667501 22957499

[CIT0025] Magnusson, L., Shangali, H.G. & Ahlström, G., 2016, ‘Graduates’ perceptions of prosthetic and orthotic education and clinical practice in Tanzania and Malawi’, *African Journal of Disability* 5(1), 142. 10.4102/ajod.v5i1.142 28730039 PMC5433444

[CIT0026] McDonald, C.L., Kartin, D. & Morgan, S.J., 2020, ‘A systematic review in prosthetics and orthotics education research’, *Prosthetics and Orthotics International* 44(3), 116–132. 10.1177/0309364620912642 32301371

[CIT0027] Ministry of Health and Social Services, 2001, *Policy on orthopaedic technical services*, Directorate: Primary Health Care, Windhoek.

[CIT0028] Ministry of Health and Social Services, 2018, *Annual report*, Directorate of Policy and Planning, Windhoek.

[CIT0029] Mwetulundila, P.K., 2019, ‘Gender equity and career progression in the Ministry of Health and Social Services in Khomas region of Namibia’, Doctoral dissertation, University of Namibia, Windhoek.

[CIT0030] O’Keeffe, B. & Rout, S., 2019, ‘Prosthetic rehabilitation in the lower limb’, *Indian Journal of Plastic Surgery* 52(1), 134–143. 10.1055/s-0039-1687919 31456622 PMC6664837

[CIT0031] O’Leary, Z., 2021, *The essential guide to doing your research project*, 4th edn., Sage Publications, London.

[CIT0032] Palinkas, L.A., Horwitz, S.M., Green, C.A., Wisdom, J.P., Duan, N. & Hoagwood, K., 2016, ‘Purposeful sampling for qualitative data collection and analysis in mixed method implementation research’, *Administration and Policy in Mental Health* 42(5), 533–544. 10.1007/s10488-013-0528-y PMC4012002 24193818

[CIT0033] Peta, C., 2017, ‘Disability is not asexuality: The childbearing experiences and aspirations of women with disability in Zimbabwe’, *Reproductive Health Matters* 25(50), 10–19. 10.1080/09688080.2017.1331684 28784066

[CIT0034] Pienaar, E. & Visagie, S., 2019, ‘Prosthetic use by persons with unilateral transfemoral amputation in a South African setting’, *Prosthetics and Orthotics International* 43(3), 276–283. 10.1177/0309364619825891 30730264

[CIT0035] Ramstrand, N., Fatone, S., Dillon, M.P. & Hafner, B.J., 2020, ‘Sharing research data’, *Prosthetics and Orthotics International* 44(2), 49–51. 10.1177/0309364620915020 32312188 PMC7178147

[CIT0036] Smith, E.M., Ebuenyi, I.D., Kafumba, J., Jamali-Phiri, M., Munthali, A. & MacLachlan, M., 2022, ‘Network analysis of assistive technology stakeholders in Malawi’, *Global Health Action* 15(1), 2014046. 10.1080/16549716.2021.2014046 35107410 PMC8812727

[CIT0037] Smith, R.O., Scherer, M.J., Cooper, R., Bell, D., Hobbs, A.D., Pettersson, C. et al., 2018, ‘Assistive technology products: A position paper from the first global research, innovation, and education on assistive technology (GREAT) summit’, *Disability and Rehabilitation: Assistive Technology* 13(5), 473–485. 10.1080/17483107.2018.1473895 29873268

[CIT0038] Trafford, Z. & Swartz, L., 2023, ‘The care dependency grant for children with disabilities in South Africa: Perspectives from implementation officials’, *Development Southern Africa* 40(2), 259–272. 10.1080/0376835X.2021.1981250 36937539 PMC7614334

[CIT0039] Urva, M., Donnelley, C.A., Challa, S.T., Haonga, B.T., Morshed, S., Shearer, D.W. et al., 2023, ‘Transfemoral amputation and prosthesis provision in Tanzania: Patient and provider perspectives’, *African Journal of Disability* 12, 1084. 10.4102/ajod.v12i0.1084 36876024 PMC9982473

[CIT0040] Vaismoradi, M., Jones, J., Turunen, H. & Snelgrove, S., 2016, ‘Theme development in qualitative content analysis and thematic analysis’, *Journal of Nursing Education and Practice* 6(5), 100–111. 10.5430/jnep.v6n5p100

[CIT0041] World Bank, 2024, *Namibia*, viewed 19 April 2024, from https://www.worldbank.org/en/country/namibia.

[CIT0042] World Health Organization, 2017, *WHO standards for prosthetics and orthotics*, WHO, Geneva.

[CIT0043] World Health Organization, 2022, *Global report on assistive technology*, viewed 09 July 2024, from https://www.who.int/teams/health-product-policy-and-standards/assistive-and-medical-technology/assistive-technology/global-report-on-assistive-technology.

[CIT0044] Wyss, D., Lindsay, S., Cleghorn, W.L. & Andrysek, J., 2015, ‘Priorities in lower limb prosthetic service delivery based on an international survey of prosthetists in low- and high-income countries’, *Prosthetics and Orthotics International* 39(2), 102–111. 10.1177/0309364613513824 24335154

